# First scientific report of a new derivative of street heroin in east of Iran

**DOI:** 10.1186/2008-2231-21-48

**Published:** 2013-06-15

**Authors:** Reza Afshari, Jamshid Tabeshpour

**Affiliations:** 1 Addiction Research Centre, Mashhad University of Medial Sciences, (Ibn-e-Sina street, Imam Reza Hospital), Mashhad 9137913316, Iran

## Dear Editor

Drugs and chemicals are easily available in Iran. Therefore, drug abuse and addiction are common [1]. Iran is a significant consumer of opium, and generally of opioid derivatives in the world [2].

Street drugs are being adulterated with lead, steroids, thallium and … for economical benefits [3,4]. This will lead to further complications. Development of tolerance to opioids in addicts leads to increase the dose or abusing more potent drugs or more harmful routes such as injection. More potent drugs are occasionally launched into the markets [5]. Opioid transitional illicit market is developing very fast. The pattern of usage is moving towards more high risk behaviors including using more potent agents and injections as well as newly developed drugs [6,7].

Coal heroin has been in use in Iran for over 50 years. In 2005, we reported crystal heroin for the first time, which is more potent and it is possible to dissolve in water in room temperature [5,8]. It relatively generates less odors in comparison to coal heroin. Recently, we encountered a new type of highly potent heroin, which took noticed by local public media [9].

Recently a new type of heroin opiate has been introduced to heavy abusers in north east Iran Mashhad. According to the clinical features of the addicts that have been referred to Medical Toxicology Centre, Imam Reza Hospital, the euphoric effects of this opiate is reported to be more potent than coal heroin, crystal heroin and crack heroin. It is called ‘’Se Doodi” (Persian;سه دودی ). It costs 2 to 2.5 times more than crystal heroin.

The clinical features are the same as heroin use including sedation, euphoria and myosis. In overdose, a decrease in consciousness level, bradypnea and pinpoint myosis is being observed. Withdrawal symptoms included restlessness, anxiety, insomnia, pain, diarrhea and sneezing. Urinary tests are positive for morphine and negative for meth/amphetamines.

This is the first report of this more potent heroin in the scientific literature. Further laboratory tests are needed to evaluate this newly introduced heroin.
